# A 12-month follow-up of a mobile-based (mHealth) obesity prevention intervention in pre-school children: the MINISTOP randomized controlled trial

**DOI:** 10.1186/s12889-018-5569-4

**Published:** 2018-05-24

**Authors:** Christine Delisle Nyström, Sven Sandin, Pontus Henriksson, Hanna Henriksson, Ralph Maddison, Marie Löf

**Affiliations:** 1 Department of Biosciences and Nutrition, Karolinska Institutet, NOVUM, 141 83 Huddinge, Sweden; 2 Department of Medical Epidemiology and Biostatistics, Karolinska Institutet, 171 77 Stockholm, Sweden; 3 Department of Psychiatry, Icahn School of Medicine at Mount Sinai, 1 Gustave L Levy Pl, New York, NY 10029 USA; 4 Seaver Autism Center for Research and Treatment at Mount Sinai, 1 Gustave L Levy Pl, New York, NY 10029 USA; 5 PROFITH “PROmoting FITness and Health through physical activity” research group, Department of Physical Education and Sports, Faculty of Sport Sciences, University of Granada, Carretera de Alfacar s/n, 18071 Granada, Spain; 6 Institute for Physical Activity and Nutrition, School of Exercise and Nutrition Sciences, Deakin University, 221 Burwood Highway, Burwood, VIC 3125 Australia; 7 Healthy Active Living and Obesity (HALO) Research Group, Children’s Hospital of Eastern Ontario Research Institute, 401 Smyth Road, Ottawa, Ontario K1H 8L1 Canada; 8 Department of Medical and Health Sciences, Faculty of Medicine and Health, Linköping University, 581 83 Linköping, Sweden

**Keywords:** Intervention, mHealth, Nutrition, Obesity, Physical activity, Pre-school, Prevention

## Abstract

**Background:**

To date, few mobile health (mHealth) interventions aimed at changing lifestyle behaviors have measured long term effectiveness. At the 6-month follow-up the MINISTOP trial found a statistically significant intervention effect for a composite score comprised of fat mass index (FMI) as well as dietary and physical activity variables; however, no intervention effect was observed for FMI. Therefore, the aim of this study was to investigate if the MINISTOP intervention 12-months after baseline measurements: (i) improved FMI and (ii) had a maintained effect on a composite score comprised of FMI and dietary and physical activity variables.

**Methods:**

A two-arm parallel randomized controlled trial was conducted in 315 healthy 4.5 year old children between January 2014 and October 2015. Parents’ of the participating children either received the MINISTOP intervention or a basic pamphlet on dietary and physical activity behaviors (control group). After 6 months, participants did not have access to the intervention content and were measured again 6 months later (i.e. the 12-month follow-up). The Wilcoxon rank-sum test was then used to examine differences between the groups.

**Results:**

At the 12-month follow-up, no statistically significant difference was observed between the intervention and control groups for FMI (*p* = 0.57) and no maintained effect for the change in composite score was observed (mean ± standard deviation for the intervention and control group: + 0.53 ± 1.49 units and + 0.35 ± 1.27 units respectively, *p* = 0.25 between groups).

**Conclusions:**

The intervention effect observed at the 6-month follow-up on the composite score was not maintained at the 12-month follow-up, with no effect on FMI being observed at either follow-up. Future studies using mHealth are needed to investigate how changes in obesity related markers in young children can be maintained over longer time periods.

**Trial registration:**

ClinicalTrials.gov (NCT02021786; 20 Dec 2013).

## Background

Overweight and obesity in childhood is a major health problem globally. In Sweden the obesity rates over the last decade have increased two-fold compared to previous decades [1]; with the prevalence of overweight and obesity in Swedish 4 year olds being between 10 to 15% [2–4]. Most well conducted obesity prevention trials which have utilized in person counselling methods have been unsuccessful in demonstrating changes on obesity markers [5–7]. Therefore, new dissemination methods such as mobile health (mHealth) programs, where the intervention is delivered using participants’ mobile phones may be of interest as they are: cost-effective, can be delivered anytime, and are interactive. In adults it has been found that mHealth programs have been effective in decreasing body weight directly after the intervention period [8, 9]. However, few studies have investigated the long term effects of mHealth interventions on lifestyle behaviors (e.g. weight management or dietary and physical activity behaviors) [10].

The Mobile-based intervention intended to stop obesity in preschoolers (MINISTOP) trial was a mHealth obesity prevention intervention, which targeted parents of 4.5 year old children, with the overall aim of improving children’s body composition, dietary habits, physical activity, and sedentary behaviors [11]. This trial included three measurement periods: baseline, the 6-month follow-up (i.e. directly after the intervention period), and the 12-month follow-up (i.e. 1 year after baseline). Between the 6- and 12-month follow-up the parents in both the intervention and control group received no support. Findings from the 6-month follow-up [12] found no statistically significant differences for fat mass index (FMI); however, an intervention effect was observed for a composite score comprised of FMI, dietary and physical activity variables. An improvement in the composite score was observed in the intervention group from baseline to the 6-month follow-up, whereas the control group showed no change (+ 0.36 ± 1.47 vs. -0.06 ± 1.33 units; *p* = 0.02 between groups). This improvement was more pronounced among children with a FMI above the median [12]. It is of interest to investigate if the change in the composite score is maintained 6 months later (i.e. the 12-month follow-up). Furthermore, even though no change was observed for FMI at the 6-month follow-up, it may be hypothesized that the changes in dietary and physical activity behaviors may result in a decreased FMI at the 12-month follow-up. Therefore, the aim of this study was to investigate if the MINISTOP intervention: (i) improved FMI and (ii) had a maintained effect on a composite score 12-months after baseline measurements.

## Methods

MINISTOP was a population based randomized controlled trial conducted in the county of Östergötland, Sweden [11] between January 2014 and October 2015. A total of 315 healthy 4.5 year old children completed baseline assessments and 156 and 159 children were randomized to either the MINISTOP intervention or control group, respectively [12]. After the 6-month intervention and at the 12-month follow-up 143 (92%) and 133 (85%) children in the intervention group and 138 (87%) and 130 (82%) children in the control group had complete measures, respectively. Informed consent was obtained from all parents. The MINISTOP trial was approved by the Research and Ethics Committee, Stockholm, Sweden (2013/1607–31/5; 2013/2250–32), and it was registered at ClinicalTrials.gov (NCT02021786; 20 Dec 2013).

Full details of the MINISTOP intervention are described elsewhere [11, 12]. Briefly, the parents of the children in the intervention group received the MINISTOP intervention, which provided an extensive program of information and behavioral support delivered via a web-based application (app) for 6 months. The MINISTOP intervention was grounded in Social Cognitive Theory [13] and included a range of theory-based behavior change techniques [14] and was based upon current guidelines for a healthy diet and physical activity patterns in pre-school children [15]. The parents were able to access the intervention content at any time and push notifications were sent regularly. The app included 12 themes, which were: healthy foods in general; breakfast; healthy small meals; physical activity and sedentary behavior; candy and sweets; fruits and vegetables; drinks; eating between meals; fast food; sleep; food outside the home; and foods at special occasions. A new theme was introduced every second week, with each theme consisting of general information, advice, and evidence-based strategies on how to change unhealthy behaviors. Parents had the ability to register their child’s intake of fruits, vegetables, candy, sweetened beverages and sedentary time within the app, and were encouraged to do so at least once per week. Parents then received graphical feedback and automated comments at the end of every week regarding their child’s registered eating and physical activity behaviors. Within the app parents had the opportunity to submit questions to a dietician and/or a psychologist to ask questions specific to their child [11, 12]. The control group received a pamphlet on healthy eating and physical activity [11, 12]. During the following 6-month period (i.e. the time after the intervention to the 12-month follow-up) no support was provided to either group. Details regarding recruitment and randomization have been published previously [12].

The primary outcome for the MINISTOP trial was FMI, while the secondary outcomes were the intakes of fruits, vegetables, candy, and sweetened beverages, as well as time spent sedentary and in moderate-to-vigorous physical activity (MVPA). In addition, as described previously [12] a composite score which included a combination of scores for FMI as well as dietary (i.e. intakes of fruits, vegetables, candy, and sweetened beverages) and physical activity variables (i.e. time spent in MVPA and sedentary) was computed for all children. Briefly, for each outcome variable the child received either a score of 1 or 0 (i.e. meeting or not meeting the pre-defined study goals which were based on relevant guidelines, respectively). The individual scores were then summed to provide a composite score which had a range between 0 and 7. The difference in the composite scores between the 12-month follow-up and baseline was calculated for each child, with a positive score difference meaning a maintained effect and a zero or negative score difference meaning no effect [12].

Body composition was assessed using the Pediatric Option for BodPod (COSMED USA, Concord, CA, USA). FMI and fat free mass index (FFMI) were calculated as fat or fat free mass (kg) divided height (m) squared [12]. Intakes of fruits, vegetables, candy, and sweetened beverages were assessed using Tool for Energy Balance in Children during 4 days. Briefly, parents took pre and post pictures of all foods and beverages their child consumed using their smartphone. A trained nutritionist then analyzed all pictures and calculated the amount of the aforementioned food groups in grams or mL per day [12, 16]. The ActiGraph wGT3x-BT accelerometer was used to assess physical activity and sedentary behavior. As described previously [12, 17], the monitor was worn on the non-dominant wrist for 7 days and the cut-points by Chandler et al. [18] were used to determine the amount of time spent sedentary and in MVPA.

The statistical methods for the trial were planned before the commencement of data collection [12]. For the purpose of this paper, analyses were conducted only on the children that provided 12-month follow-up data (i.e. a completers-only analysis). We compared the outcomes for the intervention and control group by testing the hypothesis that the two groups have equal effect in location or distribution versus the hypothesis that the two groups differ by means of the Wilcoxon rank-sum test. Exact logistic regression was used to compute the success rates between the intervention and control groups and were expressed as odds ratios and 95% confidence intervals. All analyses were conducted using SPSS version 23 (IBM, Armonk, NY, USA) using the two-sided 5% level of significance.

## Results

As shown in Table 1 there were no statistically significant differences for body composition, dietary variables, or physical activity variables between the intervention and control group at baseline for the children that completed the 12-month follow-up (*n* = 133 and 130 in the intervention and control group, respectively) (all *p*-values ≥0.08). Furthermore, there were no statistically significant differences in FMI or dietary or physical activity variables at baseline between the completers and non-completers of the 12-month follow-up (*p*-values range from 0.22 to 0.74, data not shown); which indicates that children completing MINISTOP did not differ from those that did not complete MINISTOP.

**Table 1:** Baseline characteristics of the 263 children and their parents that completed the 12-month follow-up^a^

	Intervention (*n* = 133)	Control (*n* = 130)
Children		
Sex (female), % (n)	46 (61)	47 (61)
Age (years)	4.5 ± 0.1	4.5 ± 0.2
Weight (kg)	18.3 ± 2.5	18.3 ± 2.4
Weight-for-age z-score^b^	−0.04 ± 1.09	−0.09 ± 1.04
Height (cm)	107.5 ± 4.1	107.8 ± 4.2
Height-for-age z-score^b^	− 0.05 ± 0.95	0.00 ± 0.95
BMI (kg/m^2^)^c^	15.9 ± 1.4	15.7 ± 1.2
Waist circumference (cm)^d^	53.6 ± 3.8	53.5 ± 3.4
Body fat (%)	26.4 ± 4.5	25.9 ± 4.4
FMI (kg/m^2^)	4.21 ± 0.97	4.07 ± 0.84
FFMI (kg/m^2^)	11.65 ± 0.98	11.60 ± 0.94
Fruit intake (grams/day)^e^	107 ± 72	102 ± 84
Vegetable intake (grams/day)^e^	64 ± 48	56 ± 42
Candy intake (grams/day)^e^	14 ± 18	11 ± 15
Sweetened beverage intake (ml/day)^e^	70 ± 73	56 ± 69
Sedentary time (minutes/day)^f^	480 ± 47	479 ± 54
MVPA (minutes/day)^f^	101 ± 26	100 ± 26
Parents		
Mothers’ age	36.1 ± 4.1	35.5 ± 4.4
Mothers’ BMI	24.5 ± 4.3	23.9 ± 4.2
Mothers’ education (University degree), % (n)	76 (101)	69 (89)
Fathers’ age	38.0 ± 5.1	38.1 ± 4.7
Fathers’ BMI	25.3 ± 3.2	25.4 ± 3.6
Fathers’ education (University degree), % (n)	62 (82)	55 (72)

*n* Number of children, *BMI* Body mass index, *FMI* Fat mass index, *FFMI* Fat free mass index, *MVPA* Moderate- to-vigorous-physical activity

^a^Values are provided as mean ± standard deviation unless otherwise indicated

^b^Calculated using Swedish reference data [24]

^c^Overweight and obese in the intervention group (*n* = 9, 7%; *n* = 2, 2%) and control group (*n* = 9, 7%; *n* = 1, 0.8%) [22]

^d^The number of children in the intervention and control group with waist circumference was 132 and 128, respectively

^e^The number of recording days for the dietary components was 3.9 ± 0.5 (intervention) and 3.8 ± 0.5 (control)

^f^The number of recording days for physical activity were 6.8 ± 0.8 (intervention) and 6.4 ± 1.2 (control)

Table 2 shows the differences between the 12-month follow-up and baseline measurements for body composition, dietary, and physical activity variables. No statistically significant difference was observed between the intervention and control group for FMI (*p* = 0.57) or the dietary or physical activity variables (*p*-values range from 0.10 to 0.71). However, the intervention group increased their FFMI in comparison to the control group (*p* = 0.05).

**Table 2:** Differences between the 6-month and 12-month follow-up and baseline in body composition dietary, and physical activity variables for the intervention and control group

	Difference between 6-month follow-up and baseline			Difference between 12-month follow-up and baseline		
	Intervention (*n* = 143) Mean ± SD	Control (*n* = 138) Mean ± SD	*p*-value^a^	Intervention (*n* = 133) Mean ± SD	Control (*n* = 130) Mean ± SD	*p*-value^a^
Weight (kg)	+ 1.42 ± 0.81	+ 1.26 ± 0.61	0.43	+ 2.61 ± 1.22	+ 2.34 ± 0.95	0.08
Height (cm)	+ 4.29 ± 1.08	+ 4.32 ± 1.16	0.72	+ 7.53 ± 1.61	+ 7.60 ± 1.41	0.25
FMI (kg/m^2^)	− 0.23 ± 0.56	− 0.20 ± 0.49	0.92	−0.76 ± 0.66	−0.82 ± 0.57	0.57
FFMI (kg/m^2^)	+ 0.15 ± 0.55	+ 0.01 ± 0.53	0.04	+ 0.70 ± 0.67	+ 0.56 ± 0.58	0.05
Sedentary (min/day)^b^	+ 3.6 ± 48.0	− 1.6 ± 55.0	0.18	+ 13.8 ± 51.4	+ 7.9 ± 58.4	0.22
Sedentary time (%/wear time)^b^	− 0.5 ± 4.9	−0.6 ± 5.0	0.39	−0.3 ± 5.1	−0.5 ± 5.6	0.43
MVPA (min/day)^b^	+ 9.3 ± 24.2	+ 9.8 ± 22.2	0.59	+ 14.6 ± 25.5	+ 15.8 ± 24.9	0.43
MVPA (%/wear time)^b^	+ 0.9 ± 2.8	+ 1.1 ± 2.5	0.39	+ 1.3 ± 2.8	+ 1.6 ± 2.8	0.38
Fruit (g/day)^c^	+ 2.9 ± 78.9	−12.1 ± 87.9	0.26	+ 4.3 ± 81.2	−10.0 ± 84.5	0.17
Vegetables (g/day)^c^	−6.7 ± 42.1	−3.6 ± 39.7	0.54	+ 59.5 ± 42.8	+ 51.3 ± 39.9	0.10
Candy (g/day)^c^	−0.7 ± 19.9	+ 3.1 ± 18.5	0.11	+ 1.3 ± 23.3	+ 3.9 ± 18.2	0.23
Sweetened beverages (ml/day)^c^	−12 ± 85	+ 8 ± 83	0.05	− 4 ± 100	+ 9 ± 128	0.71
Composite score^d^	+ 0.36 ± 1.47	−0.06 ± 1.33	0.02	+ 0.53 ± 1.49	+ 0.35 ± 1.27	0.25

*n* Number of children, *SD* Standard deviation, *FMI*, Fat mass index, *MVPA* Moderate-to-vigorous- physical activity

^a^Difference between intervention and control group assessed using Wilcoxon rank-sum test

^b^The number of recording days for physical activity at the 6-month follow-up and the 12-month follow-up were: 6.4 ± 1.3 days, and 6.5 ± 1.1 days (intervention) and 6.6 ± 1.0 days and 6.5 ± 1.1 days (control), respectively

^c^The number of recording days for food at the 6-month follow-up and the 12-month follow-up were: 3.7 ± 0.6 days and 3.6 ± 0.8 days (intervention) and 3.7 ± 0.6 days, and 3.7 ± 0.6 days (control), respectively

^d^Includes the scores for FMI, the intakes of fruits, vegetables, candy, and sweetened beverages, MVPA, and sedentary behavior

The composite scores at baseline for the intervention and control group were 3.53 ± 1.27 units and 3.61 ± 1.26 units (*p* = 0.48 between groups), respectively. At the 12-month follow-up, no differences in the change in the composite score was observed between the intervention and control group (+ 0.53 ± 1.49 units vs. + 0.35 ± 1.27 units, *p* = 0.25). The odds ratio for increasing the composite score for the intervention group compared to the control group was 1.26 (95% confidence interval: 0.77, 2.04; *p* = 0.36). Furthermore, no differences were observed between the groups regarding the change in the composite score for the children with a lower (≤ median) or higher (> median) FMI at baseline (*p*-value between groups = 0.62 and 0.26, respectively).

## Discussion

At the 12-month follow-up the MINISTOP intervention was not effective at reducing FMI, or in modifying dietary or physical activity variables, or the composite score. Thus the statistically significant increase in the composite score that was observed between the intervention compared to the control group at the 6-month follow-up [12] was not maintained at the 12-month follow-up. These results are consistent with two recent meta-analyses showing that childhood obesity or physical activity interventions often do not have maintained effects [19, 20]. The lack of a maintained effect for the composite score could be due to the fact that the intervention period (6-months) was not of sufficient duration or that the intervention did not adequately include behavior change strategies focused on long-term maintenance of behavior change. Increasing the duration of the intervention, allowing the parents to keep the app after the intervention, as well as modifying the intervention content to aid in retaining healthy habits may possibly help in maintaining long-term effects. The first two changes would be associated with minimal additional cost and would unlikely be associated with added burden to parents. This differs substantially from traditional face-to-face interventions where it is very difficult, time intensive, and expensive to keep group or individual counseling continuing.

Furthermore, there is a few limitations of mHealth that need to be discussed. As Vogel et al. [21] stated the limitations of using mHealth apps are established by the participants themselves. For instance, in MINISTOP the level of the parent’s technical ability might have played a role in how often they used the app, thus possibly making it more effective in those with higher technical abilities. Additionally, smartphones were not provided to the parents in MINISTOP (unless they did not own one) and therefore those with older devices might not have had as enjoyable of an experience using the app, as it might have been slower than those with newer devices. Thus, possibly causing them to use the app less and not receive as high of a dose of the intervention.

The use of other media platforms such as discussion boards would also be a good addition to the MINISTOP app in the future, as it would allow parents of children of similar ages to discuss issues or successes regarding their children’s dietary and physical activity behaviors together, anonymously. This would provide an additional support system to the parents and hopefully increase the intervention’s overall effect and retention. Additionally, it would be worthwhile to investigate if a combination of approaches, such as in-person support and a mHealth component would further increase the intervention’s effectiveness overall. This was not requested by any of the parents in the MINISTOP trial; however, possibly offering an optional monthly group session to the parents that felt like they needed more support would further aid in retaining healthy behaviors.

Interestingly, there was evidence for an increase in FFMI in the intervention group, which corresponds with the statistically significant increase found at the 6-month follow-up [12]. This indicates a positive intervention effect; however, it needs to be interpreted with caution as it could be due to a chance finding due to the multiple comparisons as no decrease in FMI or increase in MVPA were observed in the intervention group at either measurement point.

The strengths and limitations as well as the generalizability of the MINISTOP trial have been discussed previously [12]; however, it is important to highlight that MINISTOP was population-based and utilized a pre-defined statistical analytical strategy. Furthermore, at the 12-month follow-up our analyses were based only on those who completed the follow-up measures. Nevertheless, we did have high completion rates with little missing data (≥ 82% completion rate in both the intervention and control group). Therefore, there was no need to proceed with sensitivity analyses as null results were obtained. Additionally, there were no differences in baseline body composition, dietary intake, or physical activity variables for those that completed the 12-month follow-up and those that did not.

The long term effectiveness of mHealth interventions aimed at lifestyle behaviors has only been assessed in a limited number of studies to date and the majority of those studies observed no or minimal effects at or after 6-months [10]. According to Kohl et al. [10] little is known on what part of mHealth interventions are effective. The MINISTOP intervention was grounded in the Social Cognitive Theory [13] and utilized key behavior change techniques known to influence lifestyle changes [14]. However, when the MINISTOP app was created in 2013, we were unable to personalize messages specifically to each family. Many families wished this was a possibility, as many parents made this comment on the questionnaire evaluating the MINISTOP app after the intervention period. In MINISTOP families were instead grouped into three groups depending on how well they complied with the intervention using pre-defined goals. Therefore, in the future, personalization of messages that are more specific to the child or family may aid in maintaining behaviors in the long-term.

It should be noted that MINISTOP recruited participants from the general population in one Swedish county, which explains why only 28 overweight or obese children (9%) were included [12, 22]. Even though this is similar to childhood overweight and obesity rates in Sweden [23], this is a relatively low proportion of children and the parents of normal weight children might not have the same motivation to maintain or improve behavior changes in the short- or long-term. Furthermore, after the 6-month follow-up it was observed that children with a FMI above the median in the intervention group had a statistically significant better 7-component composite score than their counterparts in the control group [12]. Therefore, possibly targeting only overweight or obese children would provide a more motivated population for behavior changes.

## Conclusions

The intervention effect observed at the 6-month follow-up on the composite score was not maintained at the 12-month follow-up, with no effect on FMI being observed at either follow-up measure. Future studies using mHealth are needed to investigate how changes in obesity related markers in young children can be maintained over longer time periods.

## Abbreviations

app: Application
FFMI: Fat free mass index
FMI: Fat mass index
mHealth: Mobile health
MVPA: Moderate-to-vigorous physical activity
